# ‘So being here is. . . I feel like I’m being a social worker again, at the hospice’: Using interpretative phenomenological analysis to explore social workers’ experiences of hospice work

**DOI:** 10.1177/02692163231220163

**Published:** 2024-02-19

**Authors:** Hayley Scanlon, Gary Latchford, Matthew Allsop

**Affiliations:** 1 Leeds Teaching Hospitals NHS Trust, St James’ University Hospital, Leeds, UK; 2 University of Leeds, Leeds, England, UK

**Keywords:** Palliative care, hospices, hospice care, social workers, compassion fatigue, ‘burnout, psychological’, occupational stress, qualitative research

## Abstract

**Background:**

Social workers have a significant role in hospices working with clients who are facing death but there is limited detailed understanding of the emotional impact of this work on social workers. Research has highlighted that those involved in hospice work find the work both a struggle (e.g. because of heightened emotions) and rewarding (noting that end-of-life care can feel like a privilege).

**Aim:**

To explore UK hospice social workers’ emotional experiences of work and how this influences their practice.

**Design:**

Semi-structured interviews were conducted with hospice social workers. Interviews were transcribed and transcripts were analysed using Interpretative Phenomenological Analysis.

**Setting/participants:**

Eight social workers from different hospices in the UK.

**Results:**

Five overlapping superordinate themes emerged: making a difference to clients and families (‘the difference made’), the emotional impact of working in hospices (‘dealing with people’s emotions, and death, and dying, it’s serious stuff’), the relational context of this type of work (‘awareness of affinity to connect’), the ways in which coping is facilitated in hospices (‘seen it coming’) and a foundation theme, connection and disconnection to values (‘(dis)connection to values’).

**Conclusions:**

The results offer an exploration of social workers’ experiences of their work in hospices; how adept they were at coping and how they prepared for and made sense of the often emotionally-laden experiences encountered. Their experience of the rewards and meaning derived from their work offers important findings for clinical practice. Further research is suggested to explore a multitude of healthcare professionals’ perspectives across country settings using Interpretative Phenomenological Analysis.

**What is already known about the topic?**

- Hospice work is associated with exposure to potentially distressing situations, and the expression of empathy and compassion, which research suggests may lead practitioners to be at high risk of burnout over time.
- Social workers’ emotional experiences are largely absent from the research literature.

**What this paper adds?**

- This paper details examples of how hospice social workers make sense of aspects of the work and how they increase their resilience to the emotional impact of the work through, for example, satisfaction from helping, and reflection and awareness of the self and mortality.
- Despite the dominance of a medical model, social workers valued increased autonomy in their role in the hospice, enabling their work to align with their professional values.

**Implications for practice, theory or policy**

- Despite hospice work being associated with potentially distressing situations, social workers reported broadly positive experiences of their work.
- The work does have an emotional impact, but even in this, there were experiences of great reward, pleasure and job satisfaction.
- Unique stressors were identified, but structured support was sometimes absent; reflective spaces and clinical supervision would be beneficial in practice.

## Background

The delivery of high-quality palliative care requires interprofessional teamwork,^1^ which can change and adapt together to the needs of people with life-limiting conditions.^2^ A key component of palliative care is hospice care, a term now aligned with the care of people with life-limiting conditions.^3^ In high-resource settings such as the United States (US) and the United Kingdom (UK), the hospice model is advanced, with a requirement for the presence of a core hospice team (e.g. a hospice nurse, social worker and chaplain). Referrals can be driven by need during a progressive illness, or by prognosis (e.g. 6 months or less) in the US.^4–6^ Across settings and within interdisciplinary hospice teams, the role of the social worker is increasingly being recognised as playing a unique role in contributing to professional and nonprofessional care delivery.^7–9^ The role can include supporting the psychosocial and spiritual needs of clients and their families, formulating advanced care directives, counselling, advocacy and bereavement.^10,11^ The varied roles, skills and tasks of a hospice social worker, often not delineated, can lead to the role being perceived as ambiguous and unclear to colleagues,^12^ with boundary and role issues being a common challenge.^13^

Hospice work is associated with exposure to potentially distressing situations, coupled with expression of empathy and compassion, which may lead practitioners to be at high risk of burnout over time.^14,15^ Confronted by clients who are ill and dying, compassion may wear, resulting in ‘compassion fatigue’ or burnout.^14^ The risk of burnout was identified by Taels et al.^7^ as a potential barrier for social workers being meaningfully involved in palliative care; the emotional burden associated with the job leads to low job satisfaction (itself arising from the ‘high-stress high loss environment’). Furthermore, the work can be fast-paced due to a patient’s progressive illness, not allowing for the iterative development of long-term psychosocial treatment plans through recurrent meetings with clients and families.^16^ The risk of burnout and stress in hospice social workers is influenced by high workload, and lack of support.^17^ Research across the palliative care workforce has identified causes of stress such as ‘time-cramping’, and difficulties with boundaries.^18–20^ Protective processes are associated with satisfaction that arises from successfully working compassionately in a challenging emotional environment such as a hospice. Personal and team resilience, making a significant contribution to clients and families, and the ability to derive personal meaning from the work may all mitigate against emotional demands.^21–24^

Existing literature, largely from the US and Australia highlights the rewarding and challenging role of social work in palliative care^25^ including high levels related to stress, burnout and compassion fatigue.^26–29^ Pelon identified a need to explore in-depth the experience of the work,^29,30^ which shaped the current study. In the US, large caseloads and the primacy of physical over psychosocial aspects of care can affect interdisciplinary collaboration.^9^ However, little is known about hospice social workers’ emotional experience of their work. Research has highlighted that those involved in hospice work find the work both a struggle (for example because of heightened emotions) and rewarding (noting that social work in end-of-life care can feel like a privilege),^25^ alongside the importance of meaning derived from the work.^31^ However, there is recognition of a need to determine how to enhance worker well-being and mitigate the potential cumulative detrimental impacts of end-of-life care work.^25^ The role of vulnerability and self-care for emotional health has important implications for practice,^31^ with self-care and informal support networks, for example, able to mediate the emotional challenges of hospice social work alongside professional structures and work being ‘a calling’.^27,32,33^ ‘Compassion satisfaction’ too, appears to be a mediator for burnout and compassion fatigue.^29,34^ With emerging evidence of the stressors and protective factors experienced by social workers in a hospice environment, but relatively little qualitative investigation of the experience of the role, the present study sought to address the research question, *what are the emotional experiences of UK hospice social workers and how does this influence their practice?* Specific objectives of the research included determining what is important to hospice social workers about their work and understanding their experiences of the emotional impact of the work.

## Method

This study sought to address the research question, what are the emotional experiences of UK hospice social workers and how does this influence their practice?

### Design

A qualitative design using Interpretive Phenomenological Analysis was adopted, drawing on its theoretical and philosophical underpinnings of phenomenology, hermeneutics and idiography, allowing for exploration of the likely complexity of how participants make sense of their experiences as hospice social workers.

### Population

The target population were experienced, university-trained social workers based in UK hospices. Inclusion criteria were participants with a social work degree qualification, practising as a hospice social worker and having been working in the role for at least 3 years.

### Setting

In the UK context, most inpatient and community specialist palliative care is supported by free-of-charge, largely charity-funded hospices,^35^ including via hospice-based outpatient and day clinics, to support people with life-threatening illnesses at any stage of their disease.^36^ Services offered vary but can include inpatient care, outpatient care, care in a patient’s home or usual residence, alongside support services, counselling and therapies.

### Sample

Participants were purposively sampled social workers based in UK hospices and members of the Association of Palliative Care Social Workers (APCSW).

### Recruitment

The APCSW sent an email to all 200 members with a study information sheet. Email recipients who wanted to participate replied to the research team to express interest, with a team member (HS) making contact and scheduling an interview.

### Data collection

Semi-structured interviews were conducted by HS, a female Trainee Clinical Psychologist undertaking a doctorate in clinical psychology (DClinPsy) who had attended a weeklong training in Interpretative Phenomenological Analysis as part of the course. Participants were interviewed once over 4 months (January 2021–April 2021) via video call or telephone from home. HS had no prior relationships with participants. The participants knew HS’s occupation. Questions followed a topic guide that focused on experiences of rewards, challenges and emotional and non-clinical challenges (Appendix 1). Topics were determined through discussions with hospice social workers and engaging with the research literature to explore the experiences of, alongside rewards and causes of stress, for the palliative care and hospice workforce internationally, including social workers. This included quantitative studies focusing on stress, burnout and compassion fatigue^26–29^ and qualitative research highlighting challenges and possible mediating factors for burnout and compassion fatigue.^27,32^ Before interviews, the topic guide was pilot-tested and reviewed by an academic Interpretative Phenomenological Analysis expert to ensure questions facilitated first-person accounts. Following review, three iterations were made to the topic guide. All interviews were audio recorded and stored securely.

### Analysis

All interviews were transcribed verbatim and managed using Microsoft Word. A strategic method for doing Interpretative Phenomenological Analysis is outlined by multiple authors.^37–39^ HS flexibly followed these approaches seeking to undertake an iterative and inductive cycle which drew on specific strategies in stages^40^: listening back and familiarisation with the transcripts with a willingness to re-enter into the participant’s world; getting rid of the ‘noise’^37^ by reading transcripts and notetaking; notation in the transcript margin categorising text as descriptive, linguistic and conceptual; ‘chunked’ transcriptions highlighting when participants were talking about topics of interest; identifying emergent patterns (e.g. themes), informed by the exploratory commenting in margins; whilst identifying emergent themes returning to the highlighted coloured ‘chunks’ of a transcript to focus on a strategy for extracting themes and placing them in context, with each participant having their own themes; construct individual tables (eight tables) for participants, then moving analysis to group level, with the use of supervision from GL and MJA. A ‘dialogue’ was created between the researcher and supervisors of the coded data to think about what it means for participants to have these concerns in this working context.^41,42^ A reflective journal was kept throughout the analysis. Reporting follows the consolidated criteria for reporting qualitative research (COREQ).

### Reflexivity

The research is considered a co-production between the researcher and the participants.^43^ The authors acknowledge that their own beliefs and interests in the application of phenomenological ideas to clinical psychology and palliative care could have influenced the interpretation of the data. Clinical psychology in staff wellbeing support is an emerging area which has been heavily focused on and increasingly recognised since the pandemic. There is an overlap between social work traditions and knowledge base, however, there are distinctive qualities of clinical psychologists in terms of their focus on staff wellbeing, reflective practice and therapeutic interventions. A clinical psychology lens may have ‘opened up’ a reflective, relational and emotional focus when compared to a social work lens. HS had limited experience in the hospice setting, GL is married to a hospice social worker and MJA is an experienced researcher in palliative care. This research was completed during the COVID-19 pandemic, and this may have had an impact on the experiences participants shared.

### Ethics

Ethical approval was granted by the University of Leeds School of Medicine Research Ethics Committee on 29th October 2020 [Table table2-02692163231220163]reference number: MREC 20-001.

## Results

Invitation emails were circulated to a member list comprising 200 people, of which 17 people responded and volunteered to participate. From respondents, a sample of participants was selected for interview. Participants were aged 47 to 66 years old (mean age = 56.87 years old), with six females, and two males. Participants’ working time per week ranged from 20 to 37.5 h (*x̄* = 32 h). Hospice experience ranged from 3 to 29 years. The mean interview time was 85 min, and the range was 52 min.

### Themes of the group analysis

**Figure 1.**
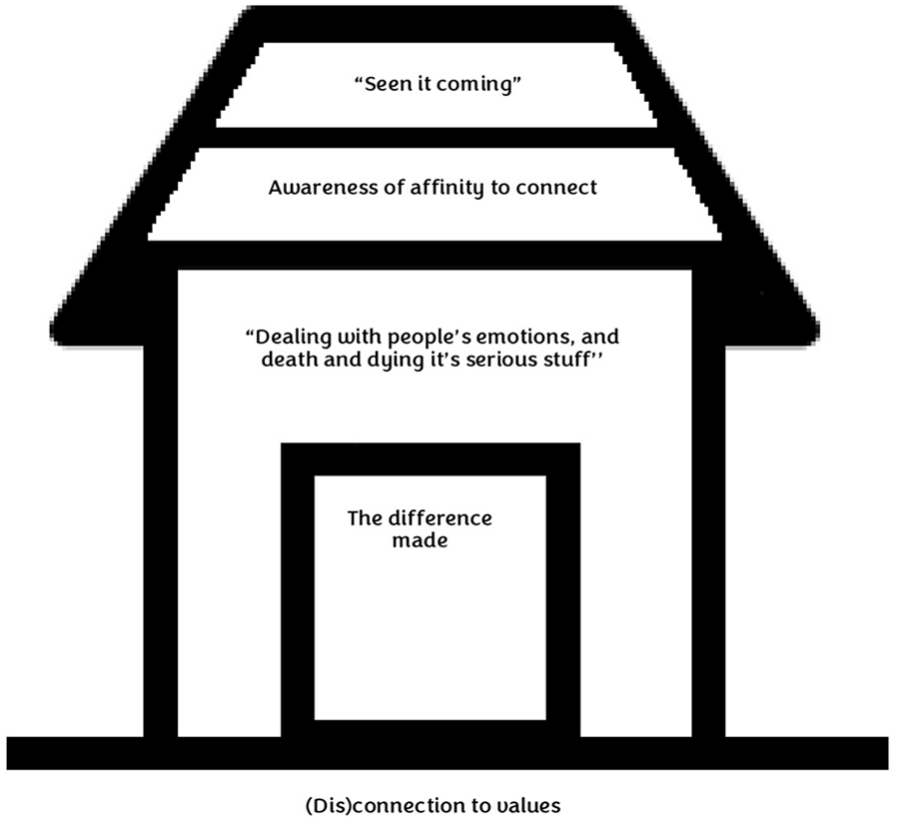
Schematic of superordinate themes. *‘The difference made’* is represented through a door whereby participants enter the house with this goal in mind; to make a difference. Participants remain in the house so long as they can engage in experiences that make a difference, ones that are perceived as satisfying, rewarding and meaningful. Inside the house participants are faced with the nature of the work – *‘dealing with people’s emotions, and death and dying it’s serious stuff’* which is represented by the interior of the house. The nature of the work is experienced as leading to a greater awareness of their own mortality, reflections on the self, matters of spirituality and how they relate to their clients. Participants’ ability to take notice of the nature of the work -the varying degree of relational complexity with clients and families is represented in the first layer of the roof – *‘awareness of affinity to connect’* encompassing and protecting the interior of the building. Participants’ ability to cope with the work with clients is represented in the top layer of the roof *‘seen it coming’* which illustrates their own coping strategies and completely covers the house protecting the building. *(Dis)connection to values* represents the foundations of the house as this theme supported participants’ resilience and ability to do the work in a meaningful way.

Analysis of accounts identified five superordinate themes; ‘the difference made’, ‘dealing with people’s emotions, and death and dying it’s serious stuff’’, ‘awareness of affinity to connect’, ‘seen it coming’ and ‘(dis)connection to values (see a schematic of themes in Figure 1 and Appendix 2 for a table with accompanying sub-themes). Participants provided feedback on the superordinate themes and sub-themes.

### The difference made

Participants felt that they were able to make a real difference in the lives of clients and families, often therapeutically, by having privileged conversations that were avoided by other professionals. Donna, discussed this was made possible by the freedom of the hospice context for social workers, and she experienced this as meaningful and rewarding, particularly when it was clear the client or family had benefitted:

“I actually am able to make a difference for people and have time with people. And for me that is really rich, you know?” (Donna, line 567).

Participants were able to have difficult conversations about death; and it seemed that even in the hospice other staff sometimes avoided them. Jericho discussed experiences of the work being rewarding and stimulating despite colleagues not taking conversations ‘further’ (Jericho, line 200). These conversations were therefore experienced as important, meaningful and rewarding:

“Being able to have that confidence about talking and with that person there about what’s gonna happen following their death” (Jericho, line 213).

As the difference they made was so integral to their experience of the work as rewarding, stimulating and freeing, it meant that it was particularly difficult if they felt unable to make a difference. For example, Luke described with vivid imagery becoming involved with a family too late, or a lack of adequate resources when he needed to refer to other services that ‘the cutbacks [had] hit’ (Luke, line 113):

“you’re confronted by situations where you could have made a difference and now you can’t. It’s a bit like, watching someone drown, and you don’t have the means to save them” (Luke, line 118).

### Dealing with death and dying – It’s serious stuff

The theme title comes from Elaine (line 359), a reminder that despite the familiarity with the work setting, death and dying are not like other issues. The social workers were working with clients and families at this crucial point in their lives, and this led to a greater awareness of their mortality and to reflections on the self and how they relate to others. Elaine explained the content of the work meant she was aware of her personal life experiences of death, and illness and how these are brought to the fore in the hospice context when working with clients:

“you have to be very aware. . . of what you’re carrying” (Elaine, line 114).

Their professional identity, investment in therapeutic social work and professional and personal values seem to have facilitated their capacity to reflect on the self. This, together with an awareness of mortality, led some to recognise a change in their appreciation of the important things in life. For Genevieve, when she reflected on her clients being given a diagnosis of a life-limiting illness or approaching death, it encouraged her to be reminded of what matters in life:

“I think we lose track, we lose track of, of what, as humans, what we need to make life . . . really meaningful” (Genevieve, line 212).

Matters of spirituality were confronted by many participants through their work, such as religion or a lack of it. For some this was an enabler in their work; for others, it led them to actively consider existential matters. Luke reflected on this specific type of work with death and dying as bringing up his unresolved spiritual matters, imagining facing his mortality and thinking about this often:

“One of the areas that we least understand, and we have to think about it a lot” (Luke, line 57).

### Awareness of affinity to connect

This theme focused on participants’ awareness of their affinity to connect with clients and families, their willingness to do this and the differing degrees to which this can happen. The emotional impact of working with clients and families presented varying degrees of relational complexity.

The degree of connection differed for participants with variations and patterns in relational connections, which was ‘just a kaleidoscope of emotions; I couldn’t even begin to untangle it you know for you’ (Luke, line 168).

For some participants, the emotional impact was clear when reflecting on relationships with clients, for example, it could be ‘challenging’ (Donna, line 359), especially after forming a strong relationship with a client who subsequently dies; ‘despite knowing a client is dying, death can still feel unexpected’ (Donna, line 394).

Jackie spoke about the nature of relationships with clients being ‘emotionally draining’ (Jackie, line 74), and explained:

“I think it’s, it’s emotionally draining because you have to be so present. In order to make that connection with somebody, you have to really give them more full attention for the time that you’re with them and really be in the moment and really try not to be distracted um and to be you know, to show them that you care and that you are trying to understand with integrity. Um and to really be empathetic. It’s hugely emotionally draining” (Jackie, line 407-411).

However, despite the emotional impact on some participants, it was not deemed unmanageable. For others, emotions were felt to be kept in check in the context of the relationship. Carson described experiences of connections with clients and families as sometimes causing her to feel *‘*sad’ (line 152), but overall found connections to be ‘so immensely satisfying and enjoyable’ (line 155–156).

Participants varied in their degree of connectedness to their clients, with a greater affinity with some. There were times when connecting to a greater degree was anticipated:

“I suppose we have had a patient of a similar age to me. . . and that is hard when you see similarities in yourself and your patient group. That is hard” (Kayla, line 17–18).

Alongside instances where it was unanticipated in the moment: ‘Some people touch you for goodness knows what reason’ (Jackie, line 102). Genevieve reflected on connecting to a greater degree with clients unexpectedly: ‘sometimes you just get hit when you don’t expect it’ (Genevieve, line 336) and, for Elaine, it ‘strikes [her] out of the blue’ (Elaine, line 42). In hindsight, Genevieve reflected it appeared to happen when she knew a client as a person through their work together, or they resonated with her own past personal experiences. For Elaine, this occurred when a client was mirroring a similar personal situation she had experienced. Participants’ ability to make sense of these varying degrees of connections appeared to be protective, guiding coping strategies.

### ‘Seen it coming’

This theme title comes from Jericho (line 243–244), illustrating the intentionality in participants’ ways of coping with their work in the hospice, particularly the direct work with clients and families, where they intentionally employed active coping. This theme envelopes the previous themes as it depicts the deliberate nature in which one copes with the nature of the work and relationships with clients. For some, their coping involved supportive relationships with colleagues and supervisors. Most could recognise the impact of their work when dealing with death and dying:

“I think ‘seen it coming’ is what’s happened. . . so seeing it coming and then reflecting at that point” (Jericho, line 243–244).

However, despite their awareness, there was a distinct lack of formal supervisory relationships. Others sought support through counselling or therapy; particularly when they recognised something in a client that reverberated with their own recent experience.

Some highlighted the importance of using professional boundaries to cope when reflecting on relationships with clients:

“Being professional and compassionate at the same time” (Genevieve, line 385).

Some participants made sense of their resilience, and how it had grown in the job. For some this was through their increasing experience of working with death and dying; others felt they had deliberately ‘built’ their resilience. Some described it as an interplay with their clients’ and families’ resilience.

The team dynamic in the hospice was described as informal and supportive, and many felt this facilitated their coping:

“It’s the about the team round about ya, knowing ya, it feels like urm, a big family, yep” (Kayla, line 105).

### (Dis)connection to values

This theme focused on participants’ felt connection and disconnection to their values. This theme is the foundation for all the themes as connectedness to values supported participants’ ability to do the work in a meaningful way. For most, it was interpreted that the hospice offered them the ability to connect with their values as a social worker and as a person, such as social justice, advocacy and compassion. They felt they had the autonomy to act within the values of the profession, which were likely personal values too:

“So being here is. . . I feel like I’m being a social worker again, at the hospice. And I’m allowed to work with people” (Donna, line 575).

“What I love about this particular hospice. . . those fundamental beliefs that I have about how social work should be is still honoured here” (Luke, line 17–18).

Many participants worked hard to broaden the perspectives of medical colleagues, in line with their values:

“They see the person as a symptom really” (Jericho, line 357).

“Making sure that um psychosocial voice is heard um and that things are really holistic and not just medical” (Jackie, line 199).

The role in the hospice was seen as offering greater autonomy than previous social worker roles in different settings, offering an opportunity to be true to their idea of the job and the values that attracted them to the profession in the first place:

“When they go to get jobs they find themselves being assessment officers, so all that training goes down the pan because they don’t end up using a fraction of it.” (Luke, lines 29–31).

Feeling connected to their values in the role seemed an important contributor to resilience in the job.

## Discussion

### Main findings

Working in end-of-life care exposes staff to potentially difficult emotions; it can be a ‘high-stress high loss environment’.^7^ The UK hospice social workers in this study were able to describe the long-term impact of their work and the way it changed them, making them confront or dwell on mortality and increasing awareness of the self. They were generally accepting and aware of their emotional experiences with clients and families, which they saw as ultimately beneficial. Psychological theories on acceptance of emotions hypothesise greater psychological flexibility when emotional experiences are accepted, rather than avoided.^44^ They readily acknowledged the emotional impact of the work but were also able to articulate rewarding experiences where they derived meaning from making a difference, which seemed to contribute to their resilience. This aligns with existing literature relating to compassion satisfaction.^32,45,46^ The satisfaction gained from helping families and having conversations about death that others avoid was reflected across participant accounts. This echoes work conducted across country settings, in which having such conversations and helping people with progressive illnesses and nearing the end of life was seen as a privilege.^25^

More surprising was that participants contrasted the role in the hospice with previous roles in social services, believing that they had much more autonomy and ability to connect with their values in the hospice. This may reflect the wider context of social work practice in the UK and multiple countries internationally, where there is a reported value crisis arising from the implementation of neoliberal and management principles, alongside reductions in welfare and social service provision.^47^ For our participants, however, this meant that they experienced their practice as much closer to their professional and personal values, providing holistic care:

‘What I love about this particular hospice, it’s not true of every hospice, is, those fundamental beliefs that I have about how social work should be, is still honoured here’ (Luke, line 17–18). This illustrates that participants’ accounts were of their organisation, reflecting that local culture could differ across individual hospices in the UK. So too could hospice leadership, which has been found to influence job satisfaction for social workers in the US.^48^ Informal support of colleagues was highly valued by participants, although structured support or supervision was largely absent in participant narratives. Reflective spaces and clinical supervision that allow for processing the emotional impact of the work could be beneficial to support practice.^49,50^

Participants also expressed frustration at the dominance of the medical model and marginalisation of psychosocial approaches even in holistic hospice care, echoed in earlier US-based research,^9^ with participants keen to raise the profile of psychosocial factors.

### What this study adds?

As hospices are an integral part of the UK health infrastructure, social workers’ emotional experiences are important in understanding how to best support and retain this workforce. Recent research has highlighted prerequisites that are key to social workers being meaningfully involved in palliative care, including enhancing the competence and confidence level of social workers, pursuing holistic and transformational social work, collaborative relationships between social workers and medical professionals, and clear role descriptions and a set of core competencies.^7^ Participants provided insights into the context surrounding these prerequisites in a UK context, including how social workers augment and supplement the work of medical professionals and the interconnectedness of professional and personal values for self-reflection. Our findings around the coping of hospice social workers may support the augmentation and development of emerging frameworks of palliative care providers’ experience and the role of resilience.^51^

Palliative care social work is seen as a vital role in the development and provision of end-of-life care services in the UK, but there is limited presence of palliative care social work at a strategic level.^52^ Research is required to explore feasible routes to enact change in this context to enhance support for hospice social workers, including countering the dominance of medical thinking and the current lack of structured professional supervision. Embedding a social work supervision model in training and education could enhance competence in coping with emotional challenges and mitigate the impact of vicarious trauma and burnout.^27^ There may also be benefits in disseminating evidence-based strategies for managing burnout, including physical activity, self-care and maintaining boundaries.^53^ Secondly, further qualitative research is needed to increase understanding of the emotional impact of hospice social work practice across country and resource contexts. We have drawn mainly on US-based literature and augmented UK evidence, but there is a need to further explore the emotional impact of palliative care social work across all settings including low-resource settings where social work is a relatively new and underdeveloped approach.^54^ This could incorporate research to explore a multitude of perspectives using Interpretative Phenomenological Analysis (multiperspectival design) that includes a range of healthcare professionals’ perspectives. Alternatively, themes could be used from this current study within a template analysis in future studies, but this would need a larger sample. Not enough studies are replicated, and this current IPA study could be replicated for future research.

### Strengths and limitations

The current study recruited eight social workers from different hospices, providing variation in experiences. Findings reflect novel perspectives of social work specific to the context of the UK and involve participants with several years of experience. Though two participants had worked as hospice social workers for only 3 years, they had qualified as social workers many years prior. All participants could contrast their hospice roles with other social work roles, but our sample reflects only a proportion of perspectives that may exist across hospice social workers. Furthermore, participants broadly reported coping well; those who volunteered to participate may have been more confident in their professional role. Recruitment through a professional network may reflect social workers who are proactive in seeking support, and selection bias may have encouraged responses from participants generally skilled at managing their well-being or who had not left the role.

## Conclusions

Hospice-based social workers are frequently engaged in emotionally demanding work, as their role is to liaise between client, family and hospice. In this study, participants appear to be coping despite being fully engaged in this work, able to articulate the impact of this work and satisfaction gained. The social work role was seen as crucial to the functioning of the hospice, linking the patient, family and hospice team. Findings from this study may reflect the organisation of hospices and the role of social workers in the UK, where hospices are mostly independent charities, though the stresses and benefits identified by participants build on earlier themes reflected across research generated mainly in the US. Alongside the need to further explore the emotional impact of palliative care social work across more country and resource settings, the absence of professional supervision and reflective spaces are areas for development to ensure support for social workers in their professional roles in the hospice.

## Appendix

**Appendix 1.** Topic guide.

**About you and your job:** 1. What sums up the work for you?	Probes, for example what was that like for you? Can you tell me a bit more? How did you feel about that? Probes, for example what are the different aspects of the role you’re doing? What’s this like for you?
2. What’s your background?	Probes, for example what has helped you in your role?
3. What were your expectations of the role?	Probes, for example were they met?
**Experience of rewards at work** 4. What stands out to you as particularly satisfying?	Probes, for example can you think of a specific worthwhile/satisfying time (extended and in-depth)
	What was that like for you? Can you tell me a bit more? How did you feel at the time? Can you think back to how you were feeling at the time? You’ve described feeling X, did you notice any other feelings? How did you feel after? What did you do after? How did it affect you? Is that typical of your experiences? In what ways was it not typical?
**Experience of challenges at work** 5. What stands out to you as particularly challenging about the work?	Probes, for example can you think of a specific challenging time (extended and in-depth)
	What was that like for you? Can you tell me a bit more? How did you feel at the time? Can you think back to how you were feeling at the time? You’ve described feeling X, did you notice any other feelings? How did you feel after? What did you do after? How did it affect you? Is that typical of your experiences? In what ways was it not typical?
6. What keeps you going? Examples of other times that have been especially emotional at work:	Probes to facilitate these into more in-depth accounts (something **different** from earlier examples, for example when working with a family was intense if family work was not mentioned much in the earlier examples)
7. Have you found the job challenging in terms of the emotional impact? (If so, how. . .)	Probes, for example can you think of a specific time that stands out? what was that like for you? Can you tell me a bit more? How did you feel at the time? Can you think back to how you were feeling at the time? You’ve described feeling X, did you notice any other feelings? How did you feel after? What did you do after? How did it affect you? Is that typical of your experiences? In what ways was it not typical?
8. How do you manage?	Probes, for example there’s a few things there that you have mentioned that are more challenging, X, X, X, how did you manage each of those? Was there a specific thing that you felt helped you cope? [Did you draw upon your own emotional resources?] Probes, for example what support do you have and what helps? what else might help? how do you make sense of that?
**Non-clinical challenges**(e.g. working as part of the team first-person examples) 9. How would you sum up your role in the hospice?	Prompts to bring out facets of interest (e.g. role ambiguity), for example times the non-clinical elements of the role have been challenging, experiences of working with other team members etc.
10. How do you think others in the team see you?	
11. What is it like as part of the hospice team?	Probes, for example anything challenging about that? What helps/would help?
**Closing down**	12. Is there anything that stands out to you most about the experiences we have spoken about? 13. Finally, anything that we have not talked about that you’d like to discuss?	

**Appendix 2. table2-02692163231220163:** Table of themes with accompanying sub-themes.

**The difference made**	‘That gives my life meaning, like doing the healing’
	Privilege to be entrusted at this time
	Talking about death as rewarding
	When not making a difference
	Striving to help and not being able to
**‘Dealing with people’s emotions, and death and dying, its serious stuff’**	Awareness of self derived from the work
	Appreciation derived from the work
	Spirituality as contemplated
**Awareness of affinity to connect**	Varying relational complexity
	‘Some people touch you for goodness knows what reason’
	Admiration and vulnerability
**‘Seen it coming’**	Active coping around connections
	Resilience as ‘built’ and an interplay
	‘A big family’
	Passive coping
**(Dis)connection to values**	Personal values
	Values of the profession
	‘They see the person as a symptom really’
	‘Charity Land’ versus financial uncertainty
	Disconnection to values of the profession
